# Recognition of DNA Lesions

**DOI:** 10.3390/ijms24119682

**Published:** 2023-06-02

**Authors:** Joanna Timmins

**Affiliations:** Institut de Biologie Structurale (IBS), University Grenoble Alpes, CNRS, CEA, IBS, F-38000 Grenoble, France; joanna.timmins@ibs.fr

The average human cell suffers from approximately 10^4^–10^5^ DNA lesions per day [1]. If left unrepaired, damaged DNA generates replication errors, mutations and genomic instability that ultimately threaten cell or organism viability and are associated with ageing and a number of human diseases, including cancer [2]. Cells have evolved several elaborate mechanisms to detect and repair DNA lesions [3,4]. These include homologous recombination (HR), non-homologous end-joining (NHEJ), global and transcription-coupled nucleotide excision repair (NER), base excision repair (BER), mismatch repair (MMR) and direct repair (DR) [4]. Tomas Lindahl, Paul Modrich and Aziz Sancar were awarded the Nobel Prize in Chemistry in 2015 for their pioneering research that led to the discovery and understanding of several of these key repair pathways [5]. For a review of the early years of DNA repair and recent advances that are not covered in this Special Issue, please consult a recent commentary by Phil Hanawalt and Joann Sweasy [6], as well as other articles published in the 50th Anniversary Special Issue of *Environmental and Molecular Mutagenesis* [7].

Regardless of the type of DNA damage, DNA repair typically occurs in four steps: (i) damage detection, (ii) damage verification, (iii) damage removal and the (iv) re-synthesis of intact DNA [3]. DNA damage recognition is a very complex cellular process that consists of detecting rare modifications to DNA in a large pool of intact genomic material, and the efficiency of this first step is critical for downstream repair processes and their consequences. This Special Issue, which includes both original research [8,9,10,11,12,13] and review articles [14,15,16,17,18,19,20], focuses on the various strategies used by cells to recognize DNA damage, but also identifies new causative agents of DNA damage, explores novel mechanisms involved in regulating DNA repair pathways, and investigates the consequences of deficient repair pathways on human health and the potential of repair factors as drug targets.

DNA lesions can be caused by both endogenous and exogenous sources. The most common endogenous sources of damage derive from the replication machinery that introduces insertions, deletions or mismatches, and reactive oxygen and nitrogen species resulting from the normal cellular metabolism. These are responsible for the generation of apurinic/apyrimidinic (AP) sites, single- and double-strand breaks (DSB), and base substitutions. The most common exogenous sources of DNA damage are ionizing and ultraviolet (UV) radiation, as well as mutagenic chemicals such as polycyclic aromatic hydrocarbons, environmental pollutants and genotoxic agents, including a number of anti-cancer drugs. In this Special Issue, Lamboy-Caraballo et al. report that stress hormones, such as norepinephrine and epinephrine, which are associated with psychological distress in some cancer patients, can also affect the integrity of genomes, notably by causing the formation of DSBs, an effect that appears to be mediated by β-adrenergic receptors [11]. Such studies will help to better understand the possible links between patient well-being and disease prognosis. In another study, Roobol et al. compared the effects of high-linear-energy-transfer (LET) α-particle radiation versus low-LET X-ray irradiation, on the formation of DSBs. They show that X-ray-induced DSBs, detected by the formation of 53BP1 foci, are quickly and more dynamically resolved than those induced by α-particle radiation. This is likely due to the high density of DSBs induced by high-LET radiation, in which DSBs are closely interspaced, leading to high local concentrations of repair proteins, which in turn modulate the efficacy of the repair process. These findings could explain the increased biological effectiveness of high-LET α-particles compared to X-ray irradiation.

Solar radiation, and in particular UV light, is a well-established source of DNA damage. Nucleotides strongly absorb UV light, especially in wavelengths ranging from 100 to 315 nm (UV-C and UV-B radiation), causing them to reach highly excited reactive states that are prone to undergoing specific photochemical reactions. This leads to the formation of cyclobutane pyrimidine dimers (CPDs), pyrimidine 6-4 pyrimidone photoproducts (6-4PPs), and their Dewar isomers. Using mechanisms of photosensitization, neighboring molecules excited by the UV light may also induce further chemical modifications to DNA. In this Special Issue, Johann de Berens and Molinier recapitulate our current understanding of the formation and recognition of such photolesions, with a special focus on the effects of genomic features and epigenetic marks on the reactivity of DNA to UV light [20]. The formation of UV-induced DNA damage is not homogeneous throughout the genome and is strongly influenced by the sequence occurrence of di-pyrimidines, the state of compaction of chromatin, and epigenetic marks such as DNA methylation.

DNA damage, however, is not restricted to DSBs and photolesions. A wide variety of DNA lesions are shown to occur in cells, ranging from small base modifications to large DNA adducts, necessitating multiple, largely distinct DNA repair mechanisms to efficiently remove lesions and maintain the integrity of the genome. Each DNA repair pathway employs distinct protein factors and strategies to recognize and remove DNA damage. This Special Issue collates three comprehensive reviews that address the key question of damage search and recognition during NHEJ, BER, and NER repair processes [15,18,19]. These reviews explore the available structural and functional data, which both shed light on the complex and dynamic mechanisms underlying damage recognition. Zahid et al. focus on the multifaceted roles of the NHEJ Ku70/Ku80 heterodimer in the recognition and repair of DSBs and in telomere maintenance [19], while D’Augustin et al. provide a thorough review of our current understanding of the mechanisms employed by the BER DNA glycosylase OGG1 to search for and find its substrate, the abundant 7,8-dihydro-8-oxoguanine (8-oxoG), within a vast excess of undamaged genomic DNA [15]. Intriguingly, D’Augustin et al. also discuss the possible scenarios that may allow DNA repair enzymes to locate rare DNA lesions amidst chromatin in the highly crowded environment of the nucleus. In contrast to these two reviews that focus on eukaryotic repair systems, Kraithong et al. compiled a review of the key recent findings regarding bacterial NER. Once more, this review illustrates the power and added value of a multidisciplinary approach combining structural, biochemical and single-molecule experiments to decipher the mechanisms that allow a small set of proteins (the Uvr proteins in bacteria) to efficiently recognize and eradicate a vast range of chemically and structurally diverse DNA lesions [18]. Importantly, all three of these reviews also address the important questions that still remain in the field and the technical challenges to overcome in exploring these complex mechanisms in vivo and in vitro.

An increasing number of recent studies have started to decipher the numerous mechanisms involved in regulating DNA repair, especially the repair of the most lethal DSBs. In this issue, one study by Dang and Morales and another by Sharma and Almasan report on the identification of novel regulators of DSB repair [9,12]. DNA replication is known to be a potent source of DNA damage and genomic instability, but Dang and Morales reveal that it may also contribute to DNA repair [12]. The loss of POLA2, a subunit of DNA polymerase alpha replication machinery increases the sensitivity of cells to ionizing radiation and PARP inhibition by favoring the accumulation of DSBs, and conversely, POLA2 overexpression in glioblastoma correlates with drug resistance and poor prognosis. Sharma et al. identified ubiquitin-specific protease 14 (USP14) as another important regulator of DSB repair pathways in response to radiotherapy [9]. Its downregulation results in increased NHEJ activity but decreased HR, suggesting that it may finetune the involvement of one or the other pathway in DSB repair.

If unrepaired, DNA damage can have severe consequences and can ultimately lead to cell death, cellular senescence or disease. Several hereditary disorders are associated with defects in DNA repair, such as ataxia telangiectasia, xeroderma pigmentosum and Cockayne syndrome [21]. The toxicity of DNA damage constitutes the underlying principle of genotoxic-based anti-cancer chemo- and radio-therapy. However, the efficient repair of such damage, notably via the BER pathway, represents a common mechanism of resistance to initially effective cytotoxic agents. Thus, the development of new-generation anticancer drugs that target DNA repair pathways is attracting increasing attention. Hans et al. present an overview of the activities of DNA glycosylases from the BER pathway in normal and cancer cells and their modes of regulation, discussing their potential as anticancer drug targets, as well as the limitations associated with such a strategy and the need for targeted inhibition [17]. DNA repair enzymes play key roles in maintaining genome integrity, and thus inhibiting these enzymes could have deleterious effects on healthy tissue. DNA damage and repair are also associated with a number of human diseases other than cancer. In this issue, for example, Hu et al. discuss the role of oxidative DNA damage and repair in atrial fibrillation and ischemic heart disease, two of the most common clinical cardiac diseases [14]. Recent evidence indicates that oxidative DNA damage might be a major underlying mechanism of cardiac diseases. Therefore, associated repair machinery also represents a potential therapeutic target for treating such diseases.

The accumulation of DNA damage in cells does not necessarily lead to disease and tumorigenesis, and cellular senescence plays an important role as a tumor suppressor mechanism. Interestingly, Hitomi et al. report that, in senescent cells, DNA damage activates the ceramide synthetic pathway, leading to an increase in the release of extracellular vesicles [10]. This in turn helps to maintain cellular homeostasis and avoid apoptosis by eliminating unwanted cytoplasmic DNA fragments derived from chromosomal DNA.

Finally, this Special Issue also includes two articles that present important developments and approaches in the study of DNA repair processes [13,16]. Inagawa et al. report the development of a novel Förster resonance energy transfer (FRET)-based assay to probe the DNA binding and release kinetics of the Ku70/Ku80 heterodimer to DNA ends [13]. This new assay allows DNA binding affinities to Ku to be measured in the context of a DNA-dependent kinase (DNA-PK) holocomplex, which paves the way for the future study of larger, physiologically more relevant NHEJ complexes to DNA ends. Das et al. developed a human papillomavirus-based model to determine both DNA replication and repair in mammalian cells and discuss the potential of this system to decipher key DNA repair mechanisms [16].

The study of DNA repair mechanisms, and more specifically of DNA damage recognition, is a very active and challenging field of research that relies on the use of complementary techniques and the development of novel, sophisticated approaches to drive its major breakthroughs. Although our understanding of DNA damage recognition has dramatically improved in recent decades, several important questions still remain unanswered. Moreover, the involvement of DNA repair in ageing and diseases, such as cancer, places it at the forefront of future research.
